# Improved motor imagery skills after repetitive passive somatosensory stimulation: a parallel-group, pre-registered study

**DOI:** 10.3389/fncir.2024.1510324

**Published:** 2025-01-07

**Authors:** Kyoko Kusano, Masaaki Hayashi, Seitaro Iwama, Junichi Ushiba

**Affiliations:** ^1^Department of Biosciences and Informatics, Faculty of Science and Technology, Keio University, Kanagawa, Japan; ^2^LIFESCAPES Inc., Tokyo, Japan

**Keywords:** motor imagery (MI), kinesthetic imagery, neuromuscular electrical stimulation (NMES), exoskeleton robot, event-related (de-) synchronization (ERD/ERS), brain-machine (computer) interface

## Abstract

**Introduction:**

Motor-imagery-based Brain-Machine Interface (MI-BMI) has been established as an effective treatment for post-stroke hemiplegia. However, the need for long-term intervention can represent a significant burden on patients. Here, we demonstrate that motor imagery (MI) instructions for BMI training, when supplemented with somatosensory stimulation in addition to conventional verbal instructions, can help enhance MI capabilities of healthy participants.

**Methods:**

Sixteen participants performed MI during scalp EEG signal acquisition before and after somatosensory stimulation to assess MI-induced cortical excitability, as measured using the event-related desynchronization (ERD) of the sensorimotor rhythm (SMR). The non-dominant left hand was subjected to neuromuscular electrical stimulation above the sensory threshold but below the motor threshold (St-NMES), along with passive movement stimulation using an exoskeleton. Participants were randomly divided into an intervention group, which received somatosensory stimulation, and a control group, which remained at rest without stimulation.

**Results:**

The intervention group exhibited a significant increase in SMR-ERD compared to the control group, indicating that somatosensory stimulation contributed to improving MI ability.

**Discussion:**

This study demonstrates that somatosensory stimulation, combining electrical and mechanical stimuli, can improve MI capability and enhance the excitability of the sensorimotor cortex in healthy individuals.

## 1 Introduction

Post-stroke hemiplegia and the associated motor impairments pose a significant burden on patients, substantially reducing the quality of life. Various motor rehabilitation methods, such as motor imagery (MI) training (Zimmermann-Schlatter et al., 2008; Di Rienzo et al., 2016; Ruffino et al., 2017; Ladda et al., 2021), mirror therapy (Altschuler et al., 1999; Rothgangel et al., 2011; Gandhi et al., 2020), and robotic-assisted rehabilitation (Bertani et al., 2017; Veerbeek et al., 2017), have been proposed, and previous studies have shown potential benefits in improving these motor impairments. However, none of these approaches has consistently shown a larger effect size compared to conventional therapy in hand motor function rehabilitation (Langhorne et al., 2009). To date, motor function rehabilitation is still a significant challenge in general.

It has been demonstrated that motor-imagery-based brain-machine interfaces (MI-BMI) can be effective for upper limb rehabilitation in post-stroke hemiplegia (Shindo et al., 2011; Ramos-Murguialday et al., 2013; Ang et al., 2014, 2015; Pichiorri et al., 2015; Cervera et al., 2018). In MI-BMI, participants perform MI and receive sensory feedback, for example, visual or somatosensory, about the change in sensorimotor cortex (SM1) excitability during MI to help them control their own SM1 excitability and therefore induce brain plasticity. To measure SM1 excitability, indexes task-related modulation of sensorimotor rhythm (SMR) derived from scalp electroencephalography (EEG) signals have been used (Pfurtscheller and Lopes Da Silva, 1999; Takemi et al., 2013, 2015). Real-time feedback about SM1 excitability can assist participants in enhancing their ability to control/regulate SM1 excitability through a trial-and-error process. As such, MI-BMI training can be described as a process in which participants learn to self-regulate the excitability of their SM1, thus promoting functional reorganization of the residual neural circuits in the brain through use-dependent plasticity (Ushiba and Soekadar, 2016).

To successfully self-regulate SM1 excitability during MI-BMI, participants search for a suitable strategy to use the BMI. Since this exploration is entirely an internal, individual process, it is essential to ensure that participants learn effective strategies that can help enhance rehabilitation effects. In particular, kinesthetic MI (KMI), which involves somatosensory imagery mimicking that of actually performing the movement, can successfully induce SM1 activation, whereas visual MI (VMI), which involves observing the movement from a third-person perspective, is less effective in relation to SM1 excitability (Stinear et al., 2006; Pfurtscheller et al., 2008; Kaiser et al., 2012; Sitaram et al., 2017) as it predominantly engages non-motor networks such as the occipital lobes but the activity is low (Neuper et al., 2005). Given that patients with severe motor disability have decreased sensitivity to kinesthetic sensations on the affected limb and are impaired in MI (De Vries and Mulder, 2007; Liepert et al., 2012), for these patients, it is crucial to ensure patients perform KMI (Takeuchi and Izumi, 2013; Marchesotti et al., 2016). However, providing participants with adequate verbal instructions and training to make them satisfactorily perform the two types of imagery (VMI and KMI) is challenging, mainly due to the implicit nature of somatosensory perception.

In this study, we test whether somatosensory peripheral stimulation performed before MI can have neuromodulatory effects and enhance subsequent MI capabilities. While somatosensory stimulation has been used as effective feedback during MI-BMI, its utility as a possible pre-conditioning component of patient training remains unclear. We conducted an experiment on healthy participants to investigate whether somatosensory stimuli, such as sensory threshold neuromuscular electrical stimulation (St-NMES) and passive movement stimulation using an exoskeleton, could aid in motor memory formation and recall, thereby improving KMI strategies. Specifically, participants were instructed to imagine the movement of opening their left (non-dominant) hand. St-NMES was applied to the left forearm Extensor Digitorum Communis (EDC) at a sensory threshold intensity that did not induce muscle contraction, helping participants become aware of the main driving muscles for hand opening. This passive movement stimulation involved an exoskeleton robot attached to the left hand, passively opening the fingers without voluntary muscle contraction, and stimulating sensory fibers through passive muscle extension. We hypothesized that using these two types of sensory stimuli would enhance joint position sense and proprioception in healthy individuals, aiding in motor memory formation and recall, thereby improving KMI performance.

## 2 Materials and methods

### 2.1 Participants

Eighteen healthy right-handed participants were recruited and data from sixteen participants were analyzed (age 18–26, 3 females, 13 males). One participant withdrew from the experiment and another one was excluded because of high baseline MI-related EEG spectral power modulation, as observed in the before-stimulation test session (see Procedure). Participants were naïve to MI practice and were categorized as right-handed based on the FLANDERS Handedness Test (all had a score ≥ 5) (Nicholls et al., 2013; Okubo et al., 2014). Participants were controlled-randomly assigned to the intervention group (*N* = 9) or control group (*N* = 7).

We had established the following exclusion criteria in advance: (1) any mental or neurological disorders, (2) any medications with psycho-neurological effects, (3) disability or pain that would interfere with movement, (4) any contraindication of a pacemaker or other device that would cause problems with electricity, (5) individuals with recorded an Event-related spectral perturbations (ERSP) value lower than −30% for the frequency band of interest, channel, and time during MI, as described below in the before-stimulation test session, (6) individuals who scored 6 or more in the Stanford Sleepiness Scale (SSS) (Hoddes et al., 1973) translated in Japanese after each test session at least once. However, no participants were excluded based on these criteria.

Participants were provided with detailed explanations about the aim and procedures of the experiments and they gave informed written consent. The experimental procedures were constructed in agreement with the Declaration of Helsinki and were approved by the ethics committee of the Department of Science and Technology, Keio University (IRB approval number: 2023–138). This randomized controlled study was conducted based on pre-registration (Open Science Framework).^1^

### 2.2 Sample size justification

To determine the appropriate sample size for this experiment, a power analysis was conducted using G*Power (version 3.1.9.6) (Faul et al., 2007, 2009), and JASP (version 0.17.2.1, JASP Team, Netherlands), R (R Core Team, 2023) based on the results of a preliminary experiment.

The preliminary experiment was initially performed with nine participants following the same time course as the main experiment. However, one participant was excluded due to inadequate task compliance, as the participant was asleep for more than half of the duration of the experiment. For the remaining eight participants, ERSP values were extracted from the C4 channel during the MI period (5–9 s) of both before-stimulation test and after-stimulation test sessions, based on each participant’s individual alpha frequency (IAF). The median values were calculated for each participant, resulting in two scalar values (before- and after-stimulation test sessions) per participant. During the before-stimulation test session, one participant with an ERSP value more than 1 SD below the group mean was excluded, as they were proficient in MI from the outset and, as such, was considered outside the scope of this study. Consequently, ERSP values from seven participants (three in the intervention group and four in the control group) were used for power analysis. The partial eta squared obtained from a two-way repeated measures ANOVA was 0.302, leading to a calculated sample size of eight participants. From this, the required number of participants to be recruited was further determined.

Given that the dropout rate due to drowsiness was 1/9, a dropout rate of 0.11 was set. Additionally, we stratified participants who were proficient in MI from the outset, defined as those with ERSP values in the before-stimulation test session lower than 50% of the group average (Schiller et al., 2010; Oyarzún et al., 2019; Nitta et al., 2020; Hodges et al., 2021). This threshold was set at −30% based on the ERSP value distribution from the preliminary experiment. Therefore, the required recruitment number was calculated as 8 / ((1–0.11) x (1–0.5)), rounded to 18 participants.

### 2.3 Procedures

**Figure 1:**
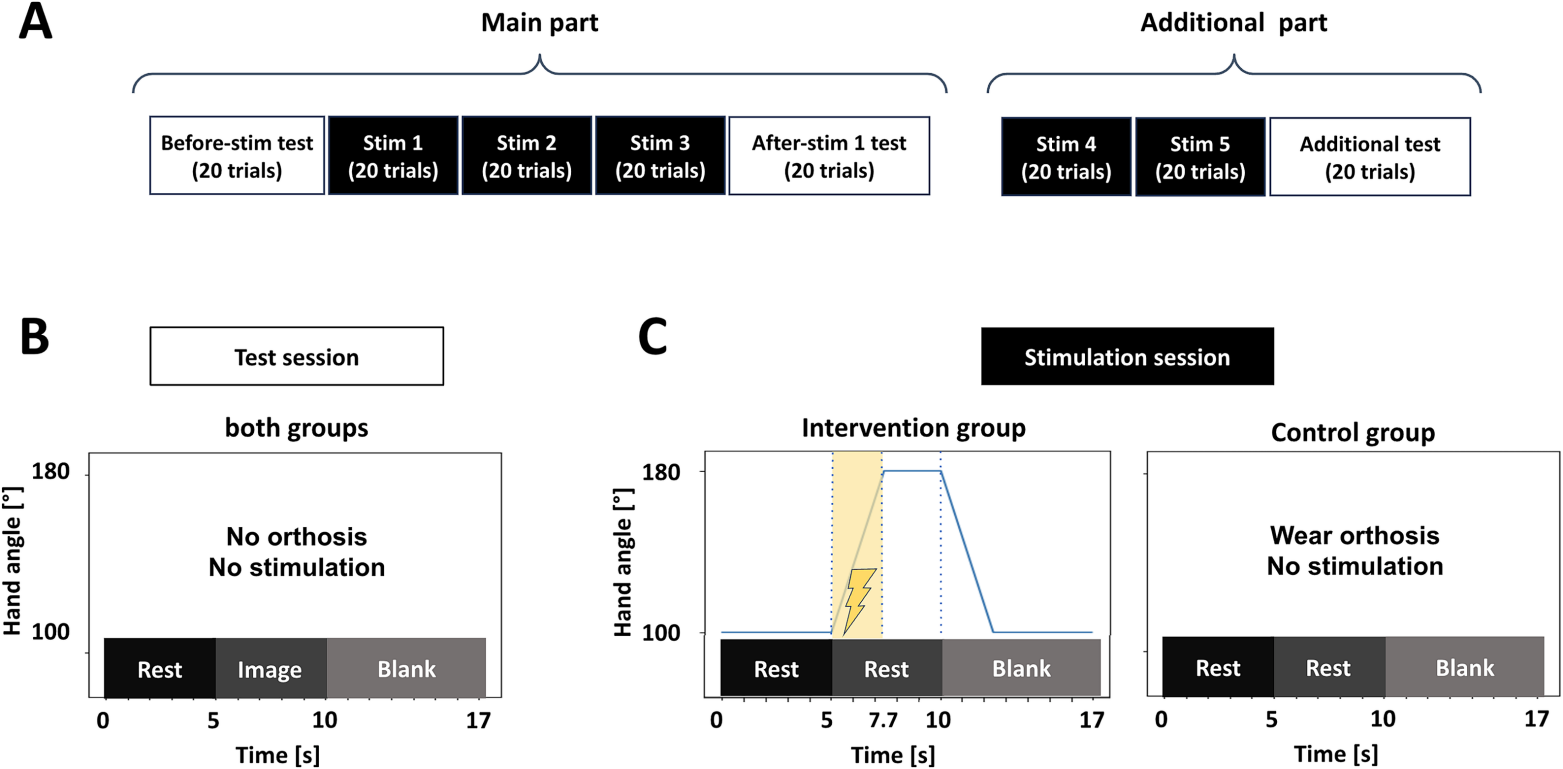
Outline of the experimental procedure. **(A)** A sequence of test and stimulation blocks. The experiment consisted of 8 sessions and lasted for 2 h. **(B)** Outline of one trial in each test session. Both groups wore no orthosis. Participants were instructed to keep rest for the first 5 s: (rest phase), then they had to perform KMI about opening their left hand for 5 s (image phase). **(C)** Outline of one trial in each stimulation session. The intervention group participants kept rest for the first 5 s (rest phase), then the orthosis opened their left hands for 2.7 s. In the meantime, the St-NMES was applied for 2.7 s. The hand was kept open for 2.3 s and closed for 2.7 s in the blank phase. The control group participants wore the same orthosis but received no stimulation during the stimulation sessions.

Participants were seated on a comfortable chair with armrests in an unshielded experimental room. A display was set up at a distance of about 1 meter in front of the chair and was used to display information about task type. The experiment consisted of two test sessions and three stimulation sessions, followed by two additional stimulation sessions and an additional test session (Figure 1A). Additional stimulation sessions and a test session were supplementally added to account for the lack of adequate stimulation time as an exploratory analysis. Only the results of the main part will be reported in this article.

A test session involved 20 trials each, with trials consisting of a 5-s rest phase (“Rest” instruction), a 5-s MI task phase (“Imagery” instruction), and a 7-s interval with no feedback (Figure 1B). The participants were instructed to remain at rest for 5 s (rest phase) and then perform a motor imagery task for 5 s, imagining opening their non-dominant left hand (task phase). Following this, they were given a 7-s break (interval). During phases other than the interval, participants were required to refrain from blinking or moving their bodies. In the stimulation sessions (Figure 1C), participants in both groups wore an orthosis on their left hand. The intervention group received somatosensory stimulation, combining electrical stimulation to the extensor digitorum communis (EDC) and passive mechanical finger extension (Figure 1C, left panel). The control group received no stimulation and remained at rest (Figure 1C, right panel). Participants in both groups repeated 20 trials per session, with the same rest, task, and interval phases, but without imagining KMI. After the three stimulation sessions, the orthosis was removed, and the after-stimulation test session was conducted. Additional stimulation sessions were performed with the same structure as the ones described above, followed by a final test session following the same procedure as in the before- and after-stimulation test sessions.

Before the experiment, participants were instructed verbally on how to execute KMI. The instruction was as follows; “When the hand opens, the EDC, a muscle that runs from the elbow to the wrist, becomes tense and contracts, splitting at the wrist to pull on the bones, muscles, and tendons of the fingers, causing the hand to open. Please imagine the sensation of this muscle contracting, as if it’s about to move just before performing the motion, yet still not exerting any force and remaining in a relaxed state.”

### 2.4 Somatosensory stimulation

Somatosensory stimulation was implemented using an exoskeleton robotic hand orthosis worn in the participant’s left hand and it was based on two types of stimuli in combination:

1. Passive movement stimulation. The robot orthosis was equipped with bars on the palmar side of the fingers, from the index to the little finger, at the second joint. These bars – one per each orthosis - were moved by a motor and allowed the fingers to open and close. During the rest phase, when the screen indicated “Rest,” the angle formed by the hand and fingers were maintained at 100 degrees, a slightly closed position. In the task phase, when the screen indicated “Task,” the hand and fingers were passively opened for 2.7 s until they reached an angle of 180 degrees, corresponding to a fully open state. For the remaining 2.3 s, the hand remained in the open state, held by the orthosis. The angular velocity of the movement was 29.6° per sec. At the onset of the blank phase, the fingers were passively closed to the initial position for 1.3 s.
2. St-NMES. The intensity of the electrical stimulation was set in advance, before the stimulation sessions in the intervention group. The intensity was discretized in 20 levels within a range of 1 to 12 mA. The electrical stimulation pulses were bipolar rectangular waves, with a pulse width of 1 ms and a frequency of 100 Hz. With the arm and hand placed palm down on a table, the location of the EDC was identified by lifting the middle finger. The electric stimulation was then applied to this area using the orthosis. The current was gradually increased from a low level until the fingertips began to move due to EDC tension or the wrist tendons became tense, and this level was designated as the motor threshold. The intensity was then reduced to about 1 mA until the participant confirmed an electrical stimulation sensation without any movement occurring and without any uncomfortable sensation, and this value was set as the stimulation intensity. During the rest phase, no stimulation was delivered. During the task phase, St-NMES was applied to the EDC for 2.7 s same as the passive movement stimulation. For the remaining 2.3 s and during the interval, there was no electrical stimulation.

### 2.5 Outcome measures

#### 2.5.1 EEG recordings

Scalp EEG signals were recorded using a 128-channel Geodesic EEG System (GES 400; Electrical Geodesics, Inc.) through a HydroCel Geodesic Sensor Net (HCGSN-128) at a sampling rate of 1 kHz. Ground and reference electrodes were placed at CPz and Cz per the extended 10–20 system, respectively. Electrode impedance was maintained below 30 kΩ. Outer channels and channels whose impedance was over 30 kΩ were excluded to ensure signal quality.

#### 2.5.2 EEG data pre-processing and analysis

The excitability of the SM1 was assessed by measuring ERD during MI tasks conducted without any feedback. To measure ERD, preprocessing was conducted using MATLAB 2023a (MathWorks, Inc., Natick, MA, USA) and Python version 3.9 (Python Software Foundation, DE, USA).

Several electrodes, specifically those on the periphery with an impedance exceeding 30 kΩ (Ferree et al., 2001), were excluded from the 129 initially available, leaving the remaining electrodes for subsequent analysis. SM1 excitability was assessed using ERSP. The process for deriving ERSP involved several steps. Initially, raw EEG signals were segmented on a trial-by-trial basis, and data from the blank phase was discarded. At this time, during the experiment, trials in which the subject visibly blinked or moved during the rest phase or the task phase were excluded as artifacts. Additionally, trials where the amplitude of the C4 electrode exceeded 50 μV (Negishi et al., 2004; De Freitas et al., 2020) during analysis were also removed due to artifact contamination. Next, this segmented signal was filtered using a bandpass filter (3–50 Hz) and a notch filter (50 Hz), both implemented through a third-order zero-phase Butterworth filter. Then, spatial filtering was performed using the common average reference (CAR) method (McFarland et al., 1997; Tsuchimoto et al., 2021). We performed CAR to eliminate the confounding effect associated with the Cz electrode due to the monopolar recording setup. The filtered signal was segmented into 1-s intervals with a 90% overlap, and a Hanning window was applied to each segment. Subsequently, SFFT (Short-term Fast Fourier Transform) was performed on each segment to obtain spectral power measurements and the ERSP was calculated based by computing the difference between rest and either imagery or task phases, as defined in this equation:

$$\mathrm{ERSP}\left(f,t\right)=\frac{A\left(f,t\right)-R\left(f\right)}{R\left(f\right)}$$

where *A(f, t)* indicates the power of the EEG signal and *R(f)* indicates the power of the reference period. In this study, *R(f)* is defined as the median power of the EEG signal from a given trial, channel, and frequency band, measured from 1 to 5 s after the onset of the “Rest” display. ERD, indicative of reduced ERSP levels (where a lower ERSP signifies an increased ERD), is linked to the excitability of the SM1 and corticospinal pathways (Takemi et al., 2013). Conversely, event-related synchronization (ERS) indicates increased ERSP levels. Additionally, ERSP was determined for specific frequency bands - alpha (IAF) and beta (IBF), which were individually identified for each participant and session. The individual IAF / IBF were chosen within the 7–14 Hz and 14–30 Hz ranges, respectively. This selection was based on identifying the continuous 3 Hz segment within these ranges that demonstrated the most pronounced average ERSP intensity during the task phase.

To construct the Time-Frequency map (T-F map), the ERSP was extracted specifically for the channel placed over the contralateral SM1 corresponding to the electrically stimulated hand. In this experiment, all the participants were right-handed, so the Channel of Interest (COI) was the C4 channel. Representative ERSP values were then taken across trials, sessions (if necessary), and participants, which were subsequently visualized as color maps in the time-frequency space. Topography maps were estimated by extracting ERSP values for each trial during the task phase, specifically in the 5 to 9-s window following the onset of rest phase. Representative ERSP values were analyzed to compute the median value across the intra-trial time, frequency (each participant’s IAF and IBF), trials, sessions, and participants, and were visualized as spatial color maps.

#### 2.5.3 Questionnaires

After each test session, three questionnaires were administered to participants, specifically:

1. The SSS was administered to exclude participants whose task compliance was low because of sleepiness (score 6 or higher). None of the participants in none of the sessions reported levels of drowsiness high enough to interfere with the experiment.
2. Two questions from the Kinesthetic and Visual Imagery Questionnaire (KVIQ) by Malouin et al. (2007). KVIQ has 20 items (10 items each in subscale: visual and kinesthetic) and two items were used in this study to assess the vividness of each dimension of MI (clarity of image/intensity of sensation), specifically one visual and another kinesthetic question about hand open movement (Tabrizi et al., 2013).
3. A Visual Analog Scale (VAS) questionnaire about subjective overall MI performance. The rightmost 0 cm point was “not at all” and the leftmost 10 cm point was “most successful.”

#### 2.5.4 Statistical analysis

Statistical analysis was performed with G*Power version 3.1.9.6 (Faul et al., 2007, 2009), JASP (version 0.17.2.1, JASP Team, Netherlands), python statsmodels version 0.13.5 (Seabold and Perktold, 2010) and scipy version 1.10.0 (Virtanen et al., 2020), R (R Core Team, 2023).

As written above, the before- and after-stimulation tests were the most effective for most participants in the preliminary experiment. Thus, we decided to use only the before- and after-stimulation test sessions as the main outcome of this study. To compare ERSP of the before- and after-stimulation test session between groups, two-way repeated measures ANOVA were utilized, followed by post-hoc pairwise t-test with Bonferroni correction. To assess possible statistically significant differences in the score of questionnaires (KVIQ and VAS) sessions between groups, the Wilcoxon signed-rank test was used.

## 3 Results

### 3.1 Stimulating effects on ERSP

**Figure 2:**
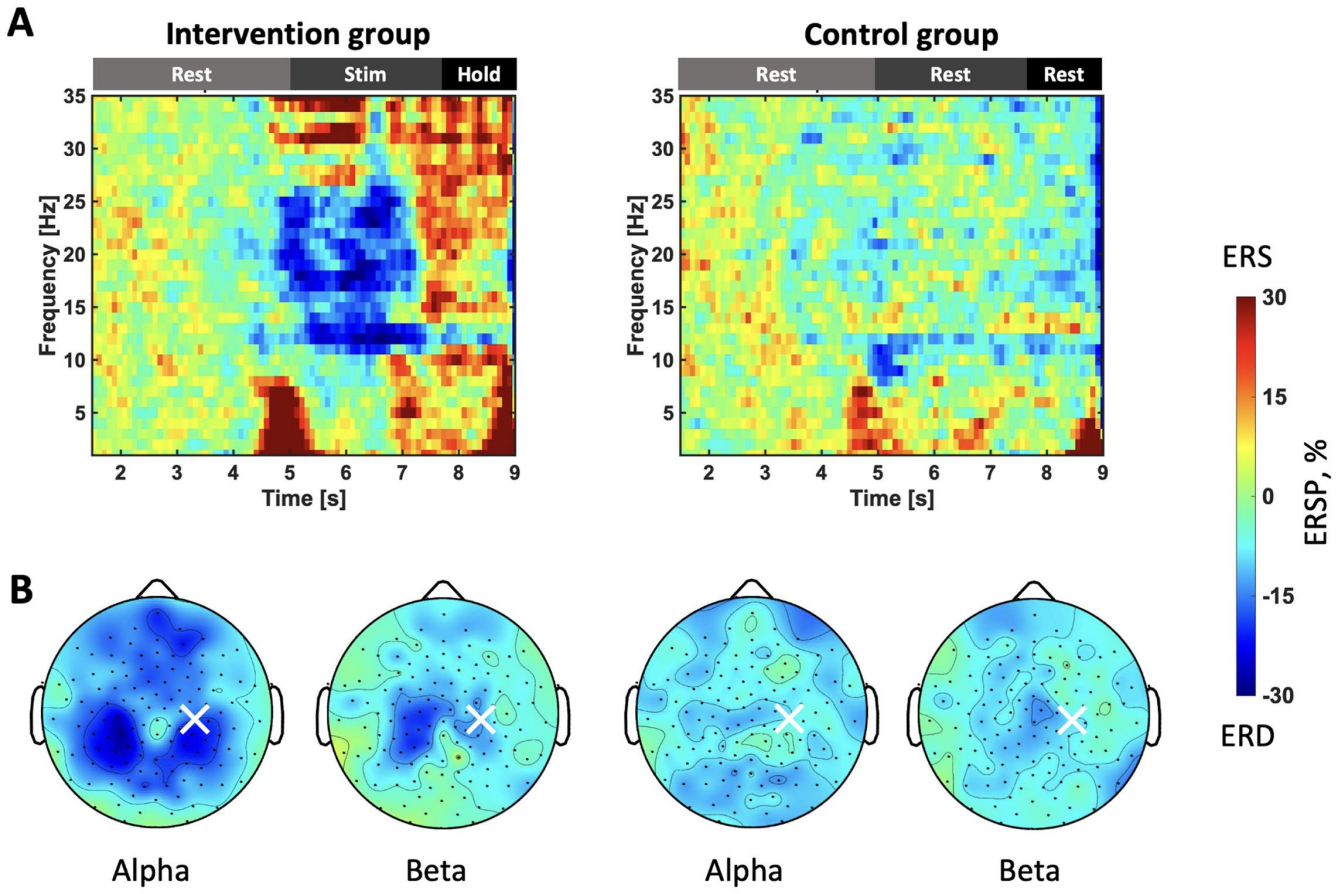
**(A)** T-F maps of the stimulation sessions in each group. The vertical axis represents the frequency of ERSP. The horizontal axis represents the number of seconds since the trial started. Before 5 s, the participants remain at rest. Stimulation is applied for the intervention group 5–7.7 s. The color indicates the intensity of the ERSP, with blue representing a stronger ERD. ERSP is standardized during the rest phase of each trial and averaged for all trials. **(B)** Topographic maps during stimulation sessions. The color bar is consistent with that in panel **(A)**. The top corresponds to the nasal side, while the bottom represents the occipital side. The ERSP of the frequency of interest (FOI), calculated for each participant, is extracted and presented as alpha and beta.

To understand the neural response to somatosensory stimulation during the resting state, we plotted T-F maps and topography maps for two conditions at the C4 electrode (Figure 2) Specifically, Figure 2A illustrates the T-F maps at the C4 channel, averaged across all stimulation trials and participants in each group. The horizontal axis represents time in seconds from the start of the rest phase. During the stimulation period (5.0–7.7 s.), a clear ERD pattern and beta rebound in the mu (11–13 Hz) and beta bands (14–25 Hz) are observed. Figure 2B displays topography maps averaged over the stimulation period (5.0–7.7 s.). The findings confirmed that ERD appeared in the ipsilateral and contralateral hemispheres in the alpha and beta rhythms only in the intervention group, and that the observed differences were significant (*p* < 0.05).

**Figure 3:**
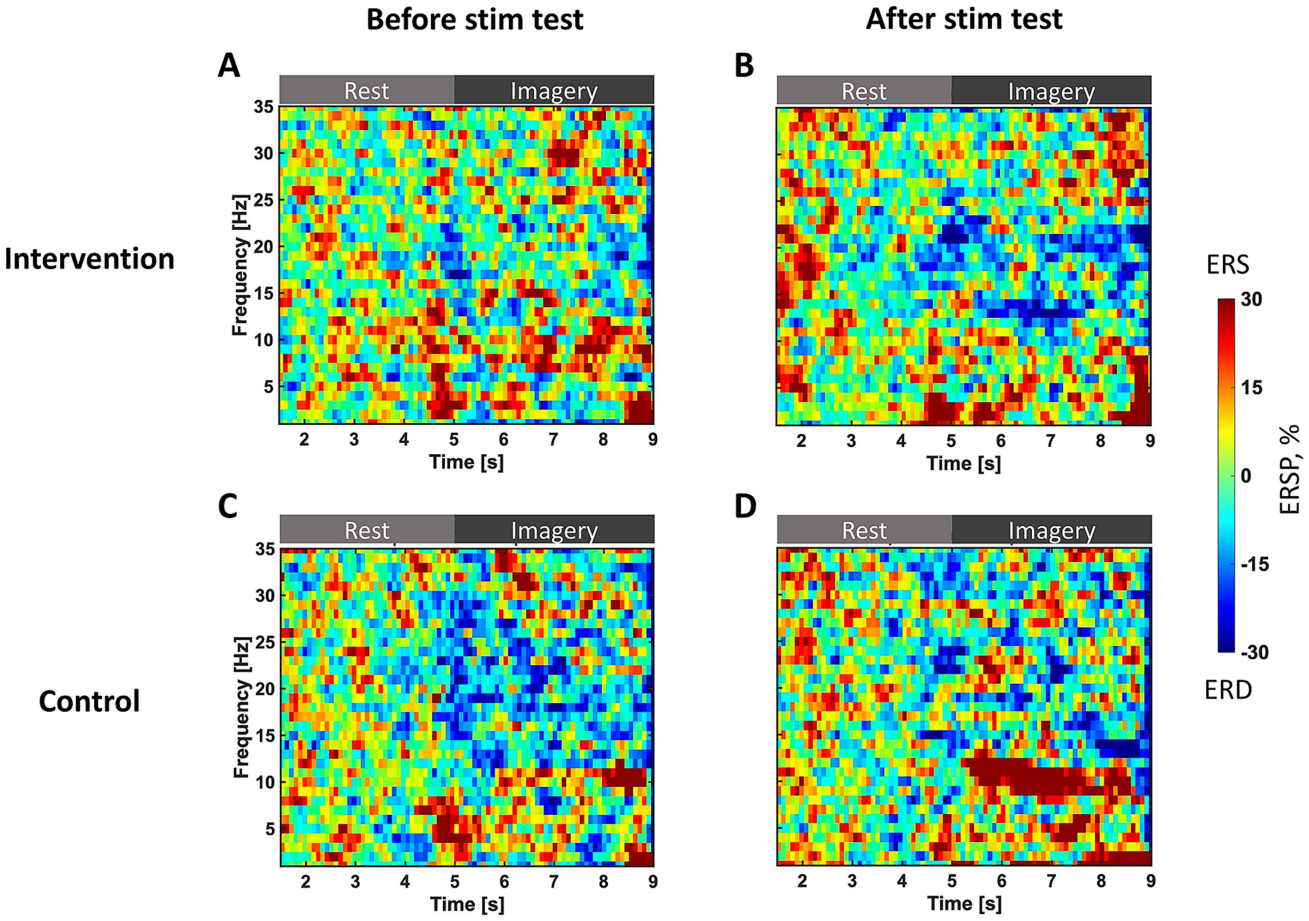
T-F maps of ERSP in the test sessions. The vertical axis represents the frequency of ERSP. The horizontal axis represents the number of seconds since the trial started. Before 5 s, the participants remain at rest; after 5 s, participants perform motor imagery of opening their left hand. ERSP is standardized during the rest phase of each trial and is averaged for all trials and in each group. The color bar represents the strength of ERSP, therefore blue indicates strong ERD. **(A)** The intervention group before stimulation and **(B)** after stimulation. **(C)** The control group before stimulation and **(D)** after stimulation.

Figure 3 presents T-F maps at the C4 channel observed in the before- and after-stimulation test sessions for both groups. The ERD was averaged across trials and participants in each group. The horizontal axis indicates the number of seconds from the start of the rest phase; before 5 s, participants remained at rest, and after 5 s, they performed MI of opening their left hand. The vertical axis represents frequency. In the intervention group, ERD is not observed during the MI phase before stimulation (Figure 3A), but after stimulation, ERD appears in the alpha (12–14 Hz) and beta (20–22 Hz) bands (Figure 3B). Conversely, in the control group, a slight ERD is observed in high beta bands (13–30 Hz) before stimulation (Figure 3C). Still, ERD after stimulation is no longer observed and ERS increases in the alpha band (Figure 3D).

**Figure 4:**
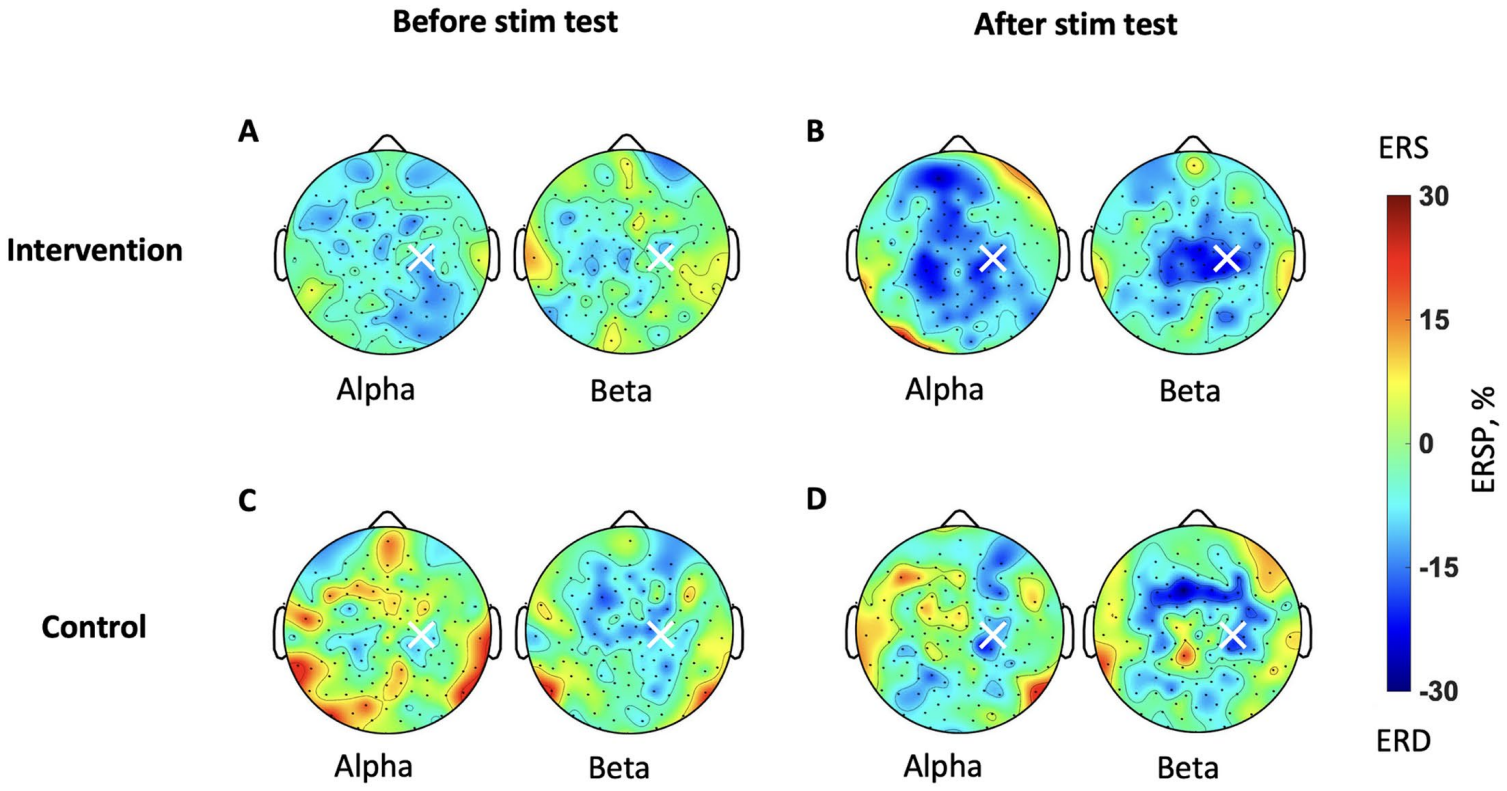
Brain topography maps during MI in the test sessions. The ERSP is averaged for each participant’s IAF and IBF during the imagery phase. The color bar represents the strength of ERSP, therefore blue indicates stronger ERD. **(A)** The intervention group before stimulation and **(B)** after stimulation. **(C)** The control group before stimulation and **(D)** after stimulation.

Figure 4 provides ERD topographic maps during MI (5–9 s in T-F maps). Before stimulation, the intervention group exhibits topographies with weak ERD over the contralateral SM1 in both alpha and beta bands (Figure 4A). After stimulation, it shows increased ERD over both contralateral and ipsilateral SM1 in both alpha and beta bands (Figure 4B). It is more focal to SM1 in the beta band than the alpha band. In the control group, exhibits topographies with less ERD over the SM1 (Figure 4C). After stimulation, it shows increased ERD mainly over contralateral SM1 in both bands (Figure 4D). Comparing both groups after stimulation, the intervention group exhibits larger ERD (Figure 4B) than the control group (Figure 4D).

**Figure 5:**
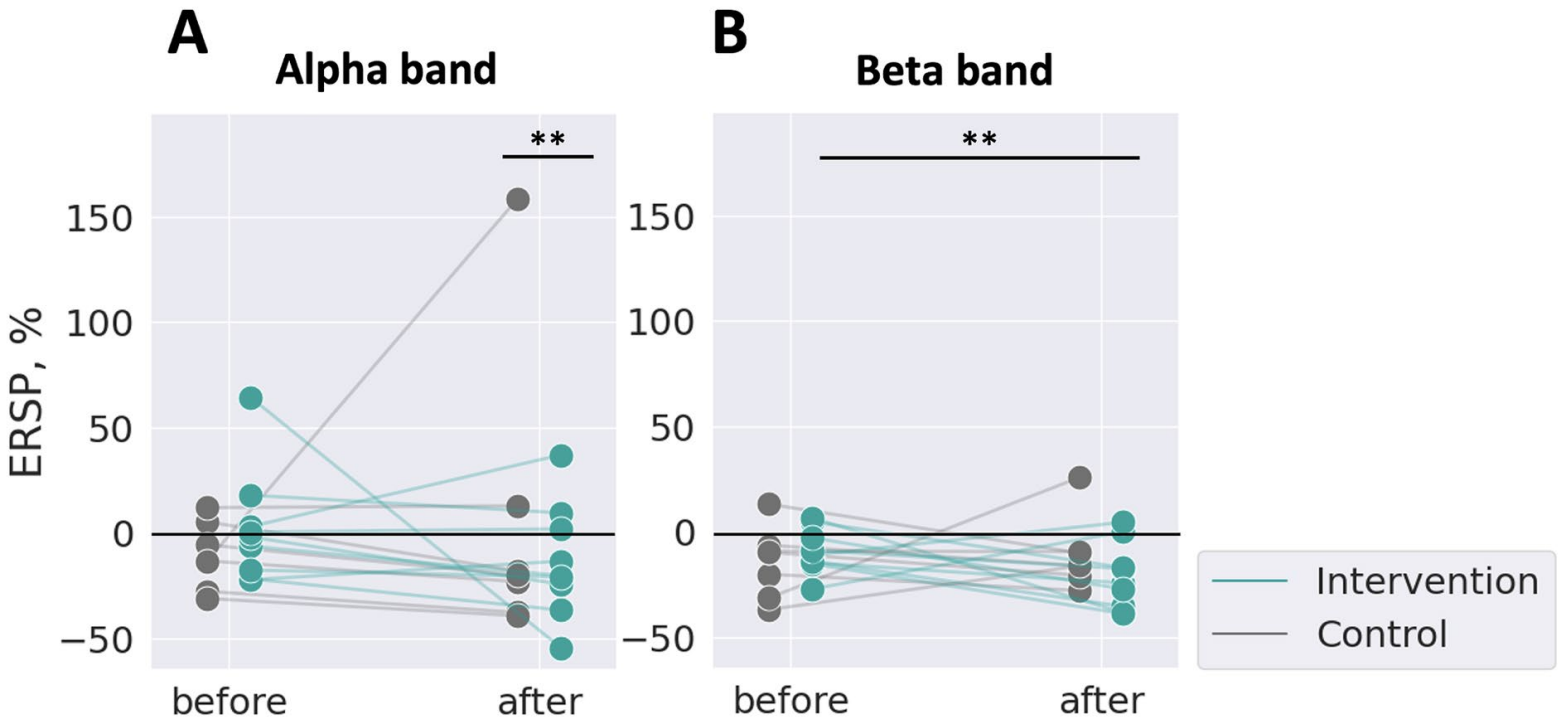
ERSP changes of IAF and IBF between the test sessions averaged for trials. One dot represents one participant. **(A)** The alpha band, **(B)** the beta band. The control group showed no effects on ERD in both bands (grey), whereas the intervention group showed a significantly increased ERD in the beta band after stimulation than before (green). **p < 0.01, with Bonferroni correction for multiple comparisons based on two-way repeated measures ANOVA.

We confirmed qualitatively that ERD during MI over SM1 was larger after stimulation than before stimulation. To quantitatively compare the level of ERD before and after stimulation between the intervention and control groups, we calculated a single scalar value per participant by averaging the ERSP values across trials in the alpha and beta bands, as shown in Figure 5. Each dot represents one participant, with green markers indicating the intervention group and grey markers indicating the control group.

A linear mixed-effects model was constructed for the alpha and beta bands with group (the intervention or the control), timing (before-stimulation test or after-stimulation test), and their interaction as fixed and random effects. The Kenward-Roger method was used to estimate degrees of freedom.

In the alpha band (Figure 5A), a two-way repeated measures ANOVA, considering the group (intervention or control) and the timing (before and after stimulation) as factors, revealed no significant main effect of the group (*F* (1, 451.13) = 2.40, *p* > 0.05) or the timing (*F* (1, 558.29) = 0.37, *p* > 0.05). However, there was a significant interaction between the group and the timing (*F* (1, 558.37) = 7.19, *p* < 0.05). Post-hoc pairwise comparisons with Bonferroni correction showed a significant difference between “control group: after stimulation” and “intervention group: after stimulation” (Estimate = 36.97, SE = 12.85, t (539.12) = 2.88, *p* < 0.0083). Other comparisons were not significant (all *p* > 0.0083). Effect sizes (partial eta-squared, η_p_^2^) were calculated for each fixed effect. The effect size for the group was small (η_p_^2^ = 0.0055), as was the effect size for the timing (η_p_^2^ = 0.00066). The interaction effect had a slightly larger effect size (η_p_^2^ = 0.013), suggesting a small but notable interaction effect.

In the beta band (Figure 5B), a two-way repeated measures ANOVA revealed a significant main effect of the timing (*F* (1, 559.31) = 6.01, *p* < 0.05). No significant main effect of the group was found (*F* (1, 217.48) = 0.12, *p* > 0.05). The interaction effect was not significant (*F* (1, 559.74) = 3.61, *p* = 0.058). Post-hoc pairwise comparisons revealed a significant difference between “intervention group: after stimulation” and “intervention group: before stimulation” (Estimate = −19.63, SE = 5.98, t (560.39) = −3.28, *p* < 0.0083). No other comparisons were significant (all *p* > 0.0083). The effect size for the group (η_p_^2^ = 0.00064) and for the interaction effect (η_p_^2^ = 0.0064) were small, but for the timing was slightly larger (η_p_^2^ = 0.011).

For ERSP, in the alpha band, a significant interaction between the group and timing was observed. In the beta band, a significant main effect of timing was identified, and post-hoc pairwise t-tests revealed a significant difference between the before and after conditions in the intervention group.

### 3.2 Stimulating effect on questionnaires

**Figure 6:**
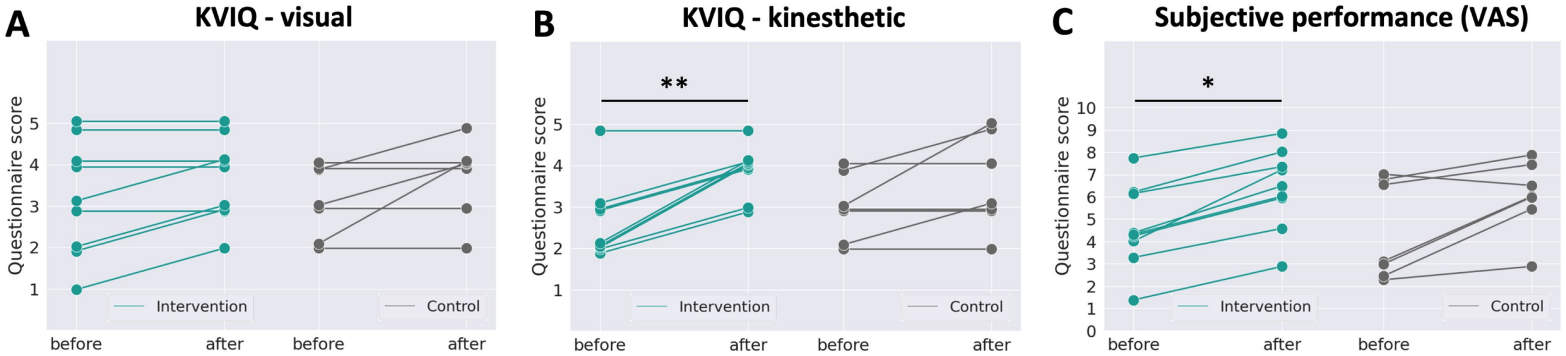
Responses to questionnaires. **(A)** The KVIQ kinesthetic question, **(B)** the KVIQ visual question and **(C)** the VAS about overall performance. The intervention group showed significant score improvement in the KVIQ kinesthetic question and the VAS after stimulation (green), but the control group did not (grey). *p < 0.05, **p < 0.01, Two-sided Wilcoxon signed-rank test.

To evaluate participants’ subjective MI skills, we analyzed the questionnaire responses; KVIQs, and VAS, as shown in Figure 6. The horizontal axis represents the sessions, and the vertical axis represents the participants’ responses. Higher scores indicate better perceived performance. In the KVIQ graphs, participants responded with a natural number of values from 1 to 5, but the overlapping values are slightly shifted for readability. The Wilcoxon signed-rank test was used to evaluate the differences between the before-stimulation test and after-stimulation test scores in the intervention group and control group. For the KVIQ Visual component (Figure 6A), no statistically significant changes between the before- and after-stimulation tests were observed in either group (intervention: *p* > 0.05; *n* = 9, control: *p* > 0.05, *n* = 7). For the KVIQ kinesthetic component (Figure 6B), the intervention group showed a statistically significant increase in score in the before- compared to the after-stimulation test (*p* < 0.01, *n* = 9), whereas no statistically significant differences were observed in the control group (*p* > 0.05, *n* = 7). For VAS (Figure 6C), the intervention group showed a statistically significant increase in score in the before- compared to after-stimulation test (*p* < 0.05, *n* = 9), whereas no statistically significant differences were observed in the control group (*p* > 0.05, *n* = 7).

## 4 Discussion

In motor rehabilitation, it has been challenging for participants to foster MI performance due to its subjective and entirely internal nature. This study investigated the neural modulation effects before and after somatosensory stimulation simulating voluntary movement in healthy individuals, while participants were at rest.

This study applied St-NMES to the EDC muscle, combined with passive movement stimulation using an exoskeleton, to naïve healthy participants at rest. We compared the ability to perform MI tasks before and after somatosensory stimulation, as well as with a control group that did not receive any stimulation. Our findings revealed that the proposed combined stimulation enhanced the excitability of the SM1 during MI.

### 4.1 Enhanced ERD after stimulation

The biomarker for SM1 excitability here used, ERD during MI, was significantly increased after somatosensory stimulation compared to before. As seen in Figure 5, the ERSP scores of the intervention and control groups were significantly different after stimulation in the alpha band, and in the beta band, the ERSP scores before and after stimulation were significantly different only in the intervention group. MI-BMI often employs SMR-ERD magnitude as neural activity indicators of sensorimotor cortex excitability (Neuper et al., 2006; Cervera et al., 2018). SMR-ERD has been shown to correlate with SM1 activity, and it is known that increased SMR-ERD is associated with heightened excitability in S1 (Babiloni et al., 1999; Miller et al., 2010; Yuan et al., 2010). These results suggest that somatosensory stimulation on the periphery had an effect on SM1 excitability in both the alpha and beta bands. Additionally, it has been reported that peripheral electrical stimulation (PES) leads to covariation in excitability not only in S1 but also in M1 (Schabrun et al., 2012). In this study, somatosensory stimulation also altered the excitability of S1, which consequently led to functional modifications in M1, thereby facilitating MI.

The potential mechanisms behind these effects can be explained in light of previous studies. Previous studies have elucidated the nature of the stimuli used in this study. One stimulus, St-NMES, activates Aβ and Aδ fibers peripherally, resulting in somatosensory perception through the activation of skin and subcutaneous nerve endings, such as Pacinian corpuscles and Merkel discs. These sensory receptors send impulses via myelinated afferent fibers to the spinal pathways, projecting directly to S1 and M1 (Maffiuletti et al., 2008). Jiang et al. (2019) reported that applying St-NMES at rest improved SM1 excitability and functional connectivity between M1 and S1, S2, and the premotor cortex (PMC). Additionally, repeated passive movement stimuli provided by a robot can activate muscle spindles and Golgi tendon organs by contracting the target muscles, which also project directly to S1 and M1. Variations similar to those observed in SMR during active movement have been reported during passive movement (Alegre et al., 2002; Lotze et al., 2003; Keinrath et al., 2006; Müller-Putz et al., 2007; Cho et al., 2011). This combination of peripheral stimuli likely reinforced motor memory formation and potentiated SM1 excitability, inducing larger ERD during subsequent MI.

### 4.2 Improvement strategy in KMI but not VMI

To assess subjective evaluations of MI strategies, we administered KVIQ and VAS to evaluate the quality of KMI, VMI, and overall performance. The results showed significant increases in KMI scores and subjective performance ratings (VAS scores) after stimulation in the intervention group, but not in the control group. The VMI scores did not significantly increase in either group, indicating that only the KMI strategy improved due to the stimulation.

MI strategies include KMI and VMI, which use independent cognitive processes (Jeannerod, 1994; Guillot et al., 2009) and engage different brain regions. KMI, which focuses attention on somatosensory sensations, activates a broad motor-related neural network, including the parietofrontal network, subcortical regions, and cerebellum, similar to motor execution and preparation. In contrast, VMI, which involves viewing movements from a third-person perspective, primarily engages the occipital and superior parietal lobules (Corbet et al., 2018). The stimulation administered in this study directly projected to SM1, a network that is active only when KMI, not VMI, is performed. As such, it may have resulted in modulating the circuits specific to KMI, not those specific to VMI, through repeated stimulation.

### 4.3 Mechanisms behind the KMI improvement from the stimuli

What role did the stimuli play in inducing KMI strategies? The MI task involved extending the fingers of the left hand and opening the hand. Intuitively, participants may focus on intrinsic hand muscles, but the primary driving muscles for this movement are extrinsic muscles like the EDC in the forearm. Hanakawa et al. emphasized the importance of acquiring accurate muscle sensations and evoking proper motor sensations for MI, noting that the quality of the motor memory is improved by stimulating limbs using congruent somatosensory afferentation (Hanakawa et al., 2008). Grush’s Emulation theory suggests that afferent feedback is involved in creating mental images of movement (Naito et al., 2002; Grush, 2004; Mizuguchi et al., 2009, 2012). Based on these studies, the somatosensory stimuli likely provided clues for accurate muscle sensations, enhancing the quality of MI. Naive participants lacked such knowledge or sensation, limiting their MI performance. However, direct stimulation to the forearm may have formed accurate muscle sensations, which could be appropriately recalled during MI.

### 4.4 Somatosensory stimulation before and during MI

This study discussed whether the quality of MI changed before and after receiving combined somatosensory stimuli of St-NMES and passive movement. Previous studies have investigated the effects of somatosensory stimuli, such as St-NMES, Mt-NMES/FES, vibrotactile, and passive movement, on MI ability. These studies generally aimed to improve BMI state discrimination accuracy or foster MI quality in participants struggling with BMI training. For example, one approach involved providing somatosensory guidance at scheduled intervals regardless of the participant’s MI quality, including St-NMES (Saito et al., 2014; Corbet et al., 2018; Vidaurre et al., 2019), Mt-NMES (Kaneko et al., 2014; Yakovlev et al., 2023), passive movement with Mt-NMES (Cho et al., 2023), and vibrotactile stimulation (Cincotti et al., 2007; Leeb et al., 2013; Ahn et al., 2014; Hehenberger et al., 2021; Ramu and Lakshminarayanan, 2023).

Another approach is to replace BMI visual feedback with somatosensory feedback when SM1 excitability increases due to motor imagery. This approach demonstrates that somatosensory feedback was more effective than visual feedback, including St-NMES (Corbet, 2019), Mt-NMES (Reynolds et al., 2015), passive movement stimulation (Ramos-Murguialday and Birbaumer, 2015; Randazzo and Iturrate, 2018). Also, the combination of somatosensory stimulation and MI enhances SM1 and corticospinal tract excitability (Kaneko et al., 2014; Saito et al., 2014; Reynolds et al., 2015; Vidaurre et al., 2019; Ramu and Lakshminarayanan, 2023). These experimental findings suggest that stimulating the sensorimotor circuits through somatosensory stimulation and performing KMI either afterwards or simultaneously may be more congruent and effective than visually guided MI in facilitating motor memory formation (Cincotti et al., 2007; Corbet et al., 2018).

While previous studies focused on providing stimuli during MI, demonstrating the superiority of somatosensory stimuli in training and motor rehabilitation effects, this study examined whether these stimuli could potentiate subsequent MI without additional stimuli. In this study, results showed increased SM1 excitability and improved subjective KMI strategy after stimulation. This is the first report to our knowledge, and this stimulation could be utilized to provide effective interventions for participants who have difficulty in increasing their SM1 excitability.

### 4.5 Potential application to BMI

This study demonstrated that administering combined somatosensory stimuli of St-NMES and passive movement to participants may enhance the quality of subsequent MI. This result has potential applications in MI-BMI based motor rehabilitation. In BMI rehabilitation, real-time feedback of SM1 excitability is provided to train participants in controlling this excitability voluntarily, inducing SM1 plasticity and promoting the recovery of motor function by reconstructing alternative damaged motor networks. Although BMI is a promising rehabilitation technology, paralyzed patients, especially post-stroke, struggle to train BMI because of a lack of proprioception (Dettmers et al., 2012) and may require long-term training (Cervera et al., 2018). The stimulation paradigm proposed in this study could offer an interesting solution to this problem. One of the reasons for this difficulty is the distortion of body image and motor engram, essential for producing and acquiring skilled movements (Monfils et al., 2005; Fuentes et al., 2013; Tosi et al., 2018). The combination of St-NMES and passive movement stimuli used in this study may help reconstruct these motor engrams. By experiencing these stimuli at rest before BMI training, the quality of MI may improve, potentially leading to successful BMI learning.

### 4.6 Limitations of this study

This study was conducted with healthy, BMI-naïve participants, and therefore the findings cannot be directly generalized to populations with different neurological conditions and responses, such as post-stroke patients with motor impairments. To address this limitation, future experiments should include individuals with paralysis in order to examine the generalizability of the results.

Another limitation is that, although participants were not informed of their group allocation, they could have easily inferred whether they belonged to the intervention or control group. To mitigate this issue, future studies could apply stimulation to muscles not directly related to the movement of interest in the control group. In the present study, the movement of interest was hand digit extension, driven by the EDC. As a potential refinement, future control groups could receive St-NMES to muscles such as the flexor digitorum superficialis (FDS), which is involved in finger flexion. Additionally, applying passive movement stimulation in the form of finger flexion movements could further improve the experimental design.

Although the results of this study were statistically significant, the effect size was not large. One possible explanation is the difference in dropout rates between the preliminary and main experiments, which suggests that the sample size may have been insufficient. Additionally, the main experiment’s effect size was smaller than that observed in the preliminary experiment. This discrepancy may be related to differences in participant characteristics. In the preliminary experiment, participants were not strictly BMI-naïve; they had prior MI-BMI training experience several months earlier. By contrast, the main experiment included strictly BMI-naïve participants. Future research should consider designing experiments based on the participant profiles used here. It is also possible that the inherently small effect size reflects a genuine characteristic of the stimulation’s impact. Future studies should investigate optimal stimulation intensities and durations to maximize efficacy. However, it is important to note that higher current intensities (e.g., Mt-NMES) can cause discomfort (Maffiuletti et al., 2008), necessitating careful adjustments. If this stimulation method is to be employed, it could serve as a pre-conditioning modality to enhance participants’ KMI strategies, potentially reducing reliance on verbal instructions. Indeed, the observed improvement in KMI clarity in the KVIQ following stimulation suggests that subsequent BMI training efficiency could be enhanced. Future studies should therefore focus on conducting BMI training after stimulation to evaluate changes in learning efficiency.

A further consideration is that the present results do not allow us to disentangle the individual contributions of St-NMES and passive movement stimulation to the observed improvements in SMR-ERD and enhanced KMI responses. Moreover, it remains speculative whether these effects stem from the electrical stimulation of nerves and muscles or from the mimicry of actual movements. While direct induction of such movements may not be essential, previous research has shown that receiving somatosensory stimulation during MI enhances SMR-ERD by increasing SM1 excitability. In our study, this enhancement was achieved solely through pre-conditioning with combined St-NMES and passive movement stimulation, without additional stimulation during the MI task. These findings highlight the novelty and potential of this approach in facilitating neural excitability and improving MI performance. Moving forward, it will be important to determine whether inducing imagined hand movements during the MI phase can further augment brain responses. Future research should examine whether the combined application of St-NMES and passive movement stimulation yields additive or synergistic benefits compared to their independent use, and which component plays a more critical role in enhancing SM1 excitability. Addressing these questions will provide valuable insights for optimizing stimulation protocols in motor rehabilitation and BMI applications.

## 5 Conclusion

This study demonstrated that combined somatosensory stimuli of St-NMES and passive motion significantly enhance kinesthetic MI quality in healthy, naïve participants. As a result, the intervention group demonstrated significantly increased ERD in the beta band. Additionally, only the intervention group reported a significant improvement in the vividness of KMI, rather than VMI, as measured by the KVIQ. The findings suggest that the somatosensory stimulation paradigm employed potentiated the SM1 and improved KMI strategy, and could be potentially further explored to develop future approaches for BMI rehabilitation.

## Data Availability

The original contributions presented in the study are included in the article/supplementary material, further inquiries can be directed to the corresponding author.

## Footnotes

^1^ https://osf.io/q3ep8
